# Musical Training and Perceptual History Shape Alpha Dynamics in Audiovisual Speech Integration

**DOI:** 10.3390/brainsci15121258

**Published:** 2025-11-24

**Authors:** Jihyun Lee, Ji-Hye Han, Hyo-Jeong Lee

**Affiliations:** 1 Hearing, Balance and Integrated Neuroscience Laboratory, Hallym University College of Medicine, Anyang 14068, Republic of Korea; jihyunlee@hallym.ac.kr (J.L.); jhhan12@hallym.ac.kr (J.-H.H.); 2 Ear and Interaction Center, Doheun Institute for Digital Innovation in Medicine (D.I.D.I.M.), Hallym University Sacred Heart Hospital, Anyang 14066, Republic of Korea; 3 Otorhinolaryngology-Head and Neck Surgery, Hallym University College of Medicine, Chuncheon-si 24252, Republic of Korea

**Keywords:** musical training, perceptual history, audiovisual information, McGurk effect, alpha power

## Abstract

Introduction: Speech perception relies on integrating auditory and visual information, shaped by both perceptual and cognitive factors. Musical training has been shown to affect multisensory processing, whereas cognitive processes, such as recalibration derived from a perceptual history, influence neural responses to upcoming sensory inputs. To investigate these influences, we evaluated cortical activity associated with the McGurk illusion focusing specifically on how musical training and perceptual history affect multisensory speech perception. Methods: Musicians and age-matched nonmusicians participated in electroencephalogram experiments using a McGurk task. We analyzed five conditions on the basis of stimulus type and participants’ responses and quantified the rate of illusory percepts and cortical alpha power between groups using dynamic imaging of coherent sources. Results: No differences in McGurk susceptibility were detected between musicians and nonmusicians. Source-localized alpha, however, revealed group-specific patterns: musical training was associated with frontal alpha modulation during integration, a finding consistent with enhanced top-down control, whereas nonmusicians relied more on sensory-driven processing. Additionally, illusory responses occurred in auditory-only trials. Follow-up analyses revealed no significant alpha modulation clusters in musicians, but temporal alpha modulations in nonmusicians depending on preceding audiovisual congruency. Conclusions: These findings suggest that musical training may influence the neural mechanisms of audiovisual integration during speech perception. Specifically, musicians appear to employ enhanced top-down control involving frontal regions, whereas nonmusicians rely more on sensory-driven processing mediated by parietal and temporal regions. Furthermore, perceptual recalibration may be more prominent in nonmusicians, whereas musicians appear to focus more on current sensory input, reducing their reliance on perceptual history.

## 1. Introduction

Understanding spoken language involves the integration of both auditory and visual cues, making speech perception a fundamentally multisensory process [1]. One compelling example is the McGurk illusion, where conflicting audiovisual speech (e.g., audio/ba/+visual/ga/) is often perceived as a fused syllable (e.g., /da/), illustrating how visual cues can override or reshape auditory perception [2]. Notably, individuals vary in their susceptibility to the illusion [3,4,5], reflecting differences in perceptual sensitivity—such as audiovisual correspondences [4,6,7], the weighting of visual cues [8,9] and the extent of integration between modalities [10]—and cognitive factors including attention [11], awareness [12], mental imagery [13], expectations [14], and perceptual recalibration based on prior sensory experience [15,16,17].

Musical training provides a valuable framework for studying variability in audiovisual integration. Musicians develop refined auditory skills, including superior pitch discrimination [18,19], improved temporal resolution [19,20], and heightened sensitivity to subtle auditory cues [21,22]. Musical training also sharpens visuospatial abilities, as musicians coordinate visual input (e.g., reading sheet music) with auditory feedback and motor actions (e.g., playing an instrument). Given this multimodal expertise, musicians may perceive audiovisual conflicts differently from untrained individuals. Some studies have reported reduced susceptibility to the McGurk illusion in trained musicians, possibly due to their heightened auditory precision [9]. Others find no difference in illusion strength [23,24]. These inconsistencies suggest that the influence of musical training on audiovisual speech integration remains unresolved, offering a promising avenue for exploring how sensory experience shapes perception.

In parallel, cognitive processes—particularly perceptual recalibration—may play a critical role in shaping multisensory perception. Perceptual recalibration refers to the brain’s adaptive adjustment of sensory processing on the basis of preceding sensory experiences, maintaining accurate and stable perception in the face of conflicting or ambiguous inputs [15]. In the context of the McGurk effect, prior exposure to audiovisual stimuli can influence subsequent perception, recalibrating the weighting of auditory and visual cues. However, previous studies have highlighted differing perspectives on the mechanisms. One suggests rapid temporal recalibration depends on the physical timing of prior audiovisual events, operating as an automatic sensory-level mechanism rather than being influenced by perceptual decisions [25]. Other shows post-perceptual decisions about orientation biased toward the opposite of preceding stimuli, balancing sensitivity and stability to optimize environmental interactions [26]. Taken together, these imply that recalibration involves both low-level sensory adjustments and higher-level decision biases. Therefore, understanding how this recalibration interacts with individual differences, such as musical training status, may provide deeper insight into the neural and cognitive mechanisms underlying multisensory speech integration.

At the neural level, audiovisual processing involves a distributed network of brain regions that resolve sensory conflicts and construct coherent perceptions [27,28]. Brain oscillations have been recognized as critical neural signatures reflecting these integrative processes [29,30,31], underpinning bottom-up and top-down processing. Bottom-up processing, linked to sensory areas such as parietal and temporal regions, integrates incoming sensory information [32,33]. Top-down processing, associated with frontal brain regions, involves attention [34,35], cognitive control [36], and expectation-driven modulation [37]. Within this framework, alpha band oscillations (8–13 Hz) are widely interpreted as markers of sensory gating and attentional modulation. Increased alpha power reflects inhibition of task-irrelevant sensory areas, enabling selective processing via top-down control [31,38]. In keeping with this perspective, musical training has been shown to influence alpha activity linked to working memory and attention, which are key cognitive functions associated with the top-down processing [39,40]. In addition, pre-stimulus alpha power can tune the quality of sensory coding and perceptual sensitivity, indicating a mechanism by which prior experience shapes upcoming processing [41].

Integrating this evidence, we hypothesized that musical training enhances top-down control during audiovisual speech processing by suppressing alpha power, thereby disinhibiting task-relevant cortical regions and increasing their activity during integration. Specifically, compared with nonmusicians, musicians would exhibit stronger alpha power decreases during audiovisual integration—both for congruent stimuli and for illusory percepts elicited by incongruent stimuli—on the McGurk task.

Initially, our focus was on perceptual and neural modulations during audiovisual integration. However, a notable rate of illusory responses during auditory-only trials prompted us to explore whether prior trial context (preceding experience) influences these responses. This observation motivated an additional hypothesis: preceding trial context (perceptual history) would shape alpha power and, in turn, influence perception on subsequent auditory-only trials. Based on enhanced top-down control associated with musical training, we hypothesized that musicians would be behaviorally less susceptible to this recalibration effect, showing less reliance on perceptual history compared to nonmusicians. Building on prior findings that adjustments to audiovisual perception following an illusory experience are represented in auditory cortex [15], we further hypothesized group-specific neural signatures: in nonmusicians, alpha power will be suppressed in auditory regions; in musicians, musical training will preferentially recruit audiovisual integration and control networks, yielding decreased frontal alpha. Accordingly, we tested whether preceding audiovisual experiences—categorized by trial congruency (congruent vs. incongruent) and perceptual response (auditory vs. illusory)—influence behavioral susceptibility and cortical alpha oscillations during subsequent auditory-only trials. By integrating behavioral outcomes and electroencephalogram (EEG) source-level alpha power, this study aims to clarify how perceptual expertise and cognitive history jointly shape multisensory speech perception on a trial-by-trial basis.

## 2. Materials and Methods

### 2.1. Participants

Thirteen musicians (6 male, mean age ± SD = 27.1 ± 5.0 years, all right-handed) and eleven age-matched nonmusicians (6 male, mean age ± SD = 26.8 ± 5.31 years, all right-handed) participated in this study. None of the participants reported taking any neurological or psychiatric medication during the six months prior to the study, and all the participants had confirmed normal or corrected-to-normal vision. The participants were informed about the tasks they would perform, the duration of the experiment, and the financial compensation provided upon completion.

All the musicians reported receiving professional musical training for more than 10 years and practicing at least three times a week during their training period. The types of musical training included vocal, piano, drum, haegeum, guitar, and violin, with detailed information provided in Table 1. The participants were recruited through online advertisements and compensated for their participation.

Both groups had normal pure-tone thresholds, with hearing loss less than 20 dB at octave test frequencies ranging from 125 to 8000 Hz, and no history of neurological or hearing disorders. The study protocol was approved by the Institutional Review Board of Hallym University Sacred Hospital, Gangwon-do, South Korea (File No. 2018-02-019-001), and written informed consent was obtained from each participant.

### 2.2. Stimuli

**Figure 1:**
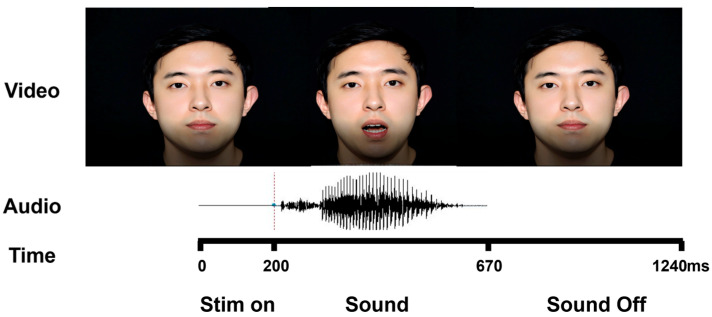
An example time window of an incongruent trial. (**Top**) three example video frames of the talker articulating /ka/ (visual). (**Middle**) waveform of the auditory track (/pa/). (**Bottom**) time axis and analysis windows. The stimulus onset is 0 ms with durations of 200 ms. The sound period extends from 200 to 670 ms, followed by a sound off period (670–1240 ms).

The stimuli were based on audio-visual recordings of the syllable /pa/ spoken by a male talker. As shown in Figure 1, the video captured his face while articulating the syllable. The audio-visual materials were recorded with a Canon EOS 6D DLSR camera (Canon, Tokyo, Japan) connected to an external Zoom F1-SP microphone (Zoom Corp., Tokyo, Japan) mounted on a tripod in a sound-attenuated booth. The audio stimuli were recorded as WAV files in stereo with a 24 bit rate and a sampling rate of 48 kHz. Audio and Video streams were synchronized and processed offline with Adobe Premiere Pro CS6 software to develop three stimuli: audio only (audio/pa/), congruent (audio/pa/with video/pa/), and incongruent (audio/pa/with video/ka/).

The total duration of the audio-only stimulus was 1240 ms, with a voice onset time (VOT) of 360 ms, during which a black screen was displayed. Similarly, the total duration of the congruent audiovisual stimulus was 1240 ms, with a VOT of 360 ms. In the incongruent stimulus condition, the voice onset time was adjusted to 200 ms to align with the first detectable lip movement frame of the video file depicting /ka/, resulting in a total duration of 1240 ms (Figure 1).

### 2.3. Electroencephalogram (EEG) Acquisition

#### 2.3.1. Experimental Procedure

The three McGurk stimuli were presented randomly using MATLAB (2024b) (MathWorks, Inc., Natick, MA, USA), with each stimulus being shown at least 100 times. Participants were seated in a comfortable reclining chair and were instructed to select one of four response options—audio, visual, fusion, and others—after perceiving the stimuli through two-channel speakers positioned 1.0 m away.

#### 2.3.2. EEG Acquisition and Data Processing

Multichannel EEG data were acquired using the actiCHamp Brain Products recording system (Brain Products GmbH, Gilching, Germany). Scalp potentials were recorded at 64 equidistant electrode sites, and all electrodes were referenced to the vertex sensor. The ground electrode was positioned above the forehead. Electrode impedances were maintained below 10 kΩ, and EEG signals were amplified and digitized at 1000 Hz. During the EEG recording, continuous data were bandpass-filtered from 0.01 to 120 Hz and a notch filter for 60 Hz noise was applied.

In the current study, all EEG data were preprocessed offline using Brain Vision Analyzer 2.2 (Brain Products GmbH, Gilching, Germany). Raw data were visually inspected, and the segments containing excessive gradients (>50 µV/ms) were marked and removed. Bad channels identified during this step were interpolated using spline interpolation. The data were then downsampled to 500 Hz and filtered with a zero-phase Butterworth filter from 0.01 to 120 Hz. An independent component analysis (ICA) algorithm was applied to reject ocular and muscle artifacts. ICA components corresponding to eye blinks, horizontal eye movements, and muscle noise were manually identified and rejected. After artifact correction, the data were downsampled to 250 Hz and re-referenced to the average of all electrodes. The data were further analyzed in MATLAB using the toolboxes FieldTrip [42] and EEGLAB [43] and with customized codes.

### 2.4. EEG Data Analysis

#### 2.4.1. Selection of Trials for Conditions

Only trials with correct responses were considered for the congruent condition, defined as trials in which participants’ responses matched the auditory syllable (Cong). In the audio-only and incongruent conditions, trials were categorized on the basis of perceptual outcomes: responses matching the auditory syllable were classified as auditory perception (Aonly-A, Incong-A), whereas responses indicating the McGurk illusion were classified as illusory perception (Aonly-I, Incong-I).

#### 2.4.2. Selection of Preceding Trials for the Current Audio-Only Condition

To investigate whether the audio-only condition was influenced by preceding trials, we selected trials in which the current condition was audio-only. We subsequently identified the immediately preceding trials that involved either congruent or incongruent audiovisual stimuli, irrespective of the participant’s response. To isolate the effect of prior audiovisual information, we excluded cases where the preceding trial was also an audio-only condition. On the basis of this selection, we categorized the current audio-only trials according to the type of preceding trial (congruent or incongruent) and the participant’s current response (auditory or illusory). This resulted in four conditions for analysis based on the combination of previous and current trial characteristics: Congruent/A-only–Auditory (Cong_T1_–A), Congruent/A-only–Illusion (Cong_T1_–I), Incongruent/A-only–Auditory (Incong_T1_–A), and Incongruent/A-only–Illusion (Incong_T1_–I).

#### 2.4.3. Time-Frequency Analysis

The data underwent an additional high-pass filter at 0.5 Hz to mitigate residual low-frequency noise. Epochs were defined to encompass the duration of stimuli, including a portion of the response time, spanning from 1 s before stimulus onset to 2 s after. Artifact rejection was performed on these epochs using amplitude thresholds set between −150 and 150 µV to exclude noise trials. The time-frequency analysis was conducted with a Hanning taper. The analysis parameters were configured as follows: power output was computed using the ‘mtmconvol’ method, and the frequency of interest ranged from 1 to 30 Hz, with a step size of 0.5 Hz. The time windows for analysis were adjusted dynamically to 3 cycles per frequency, providing a balance between temporal resolution and spectral resolution. The time of interest spanned from −1 to 2 s relative to the stimulus onset, with a step size of 4 ms. The data were padded to the next power of two to optimize computational efficiency. The analysis was applied to each condition dataset using the FieldTrip function.

#### 2.4.4. Source Analysis Using Dynamic Imaging of Coherent Sources (DICS)

**Figure 3:**
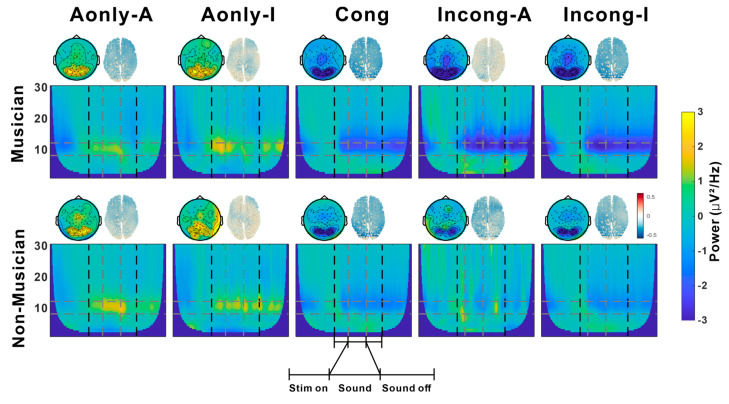
Time-frequency analysis by condition and group. Rows showing musicians (**top**) and nonmusicians (**bottom**). The columns show the five conditions. The main figures depict time-frequency representations (1–30 Hz) averaged across participants. The vertical dashed lines delineate the three analysis windows (Stim on, Sound, Sound off). The horizontal dashed lines indicate the alpha power frequency from 8 to 13 Hz. Above each panel, the left circular inset shows the change in the scalp topography of the alpha band (8–13 Hz) during the sound window. The right brain indicates source-localized alpha power using DICS during the sound window.

**Figure 4:**
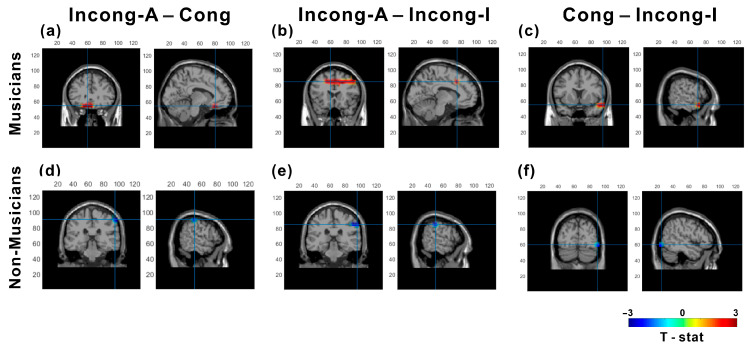
Source-level alpha (8–13 Hz) differences during the sound period. Dynamic imaging of coherent sources (DICS) contrasts are shown separately for musicians (top row; (**a**–**c**)) and non-musicians (bottom row; (**d**–**f**)). Each pair depicts clusters that survived the linear mixed-effects permutation test with cluster-based correction (*p* < 0.05). Red indicates greater relative alpha power in the first condition of each contrast; blue indicates greater power in the second. The clusters are localized primarily to frontal (**a**,**b**) and temporal areas (**c**) in musicians and to parietal (**d**,**e**) and occipital (**f**) areas in nonmusicians. Blue cross lines indicate a pick cluster.

**Figure 6:**
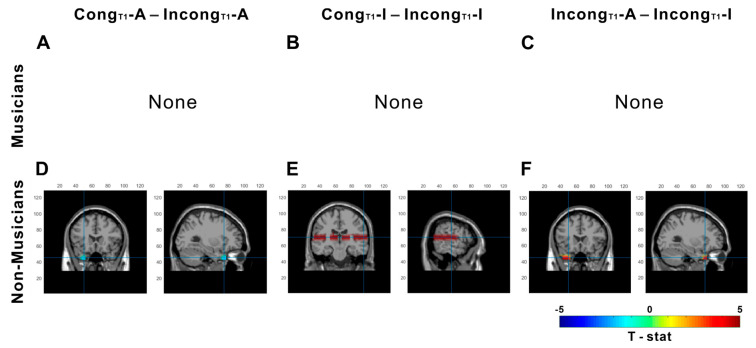
Source-level alpha (8–13 Hz) differences in auditory-only trials preceded by AV congruency during the sound period. Rows separating musicians (**A**–**C**) and nonmusicians (**D**–**F**). Each panel shows the clusters that survived the linear mixed-effect permutation tests with cluster-based correction (*p* < 0.05) during the sound period of the current auditory-only trial, contrasting pairs of conditions defined by the preceding audiovisual trial and the current response. Red indicates greater relative alpha power in the first condition of each contrast; blue indicates greater power in the second condition. No significant clusters were found (**A**–**C**) in musicians and to the temporal and parietal (**D**) and temporal (**E**,**F**) areas in nonmusicians. None denotes no significant differences for that contrast in that group. Blue cross lines indicate a pick cluster.

**Figure 7:**
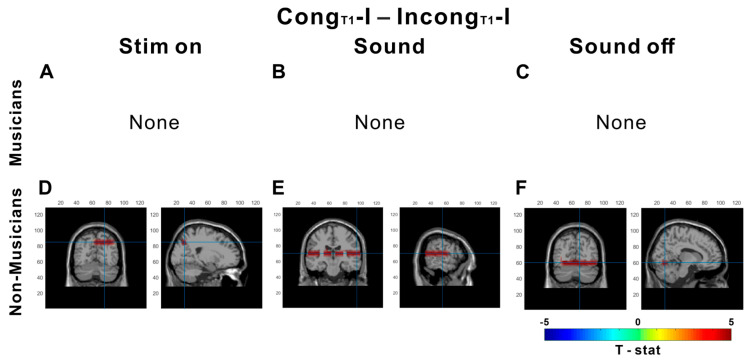
Time-resolved source-level alpha (8–13 Hz) differences for auditory-only trials as a function of prior AV congruency. Rows separating musicians (**A**–**C**) and nonmusicians (**D**–**F**). The columns show three analysis windows of the current auditory-only trial: stimulus onset, sound, and sound off. Maps display clusters from a DICS beamformer analysis of alpha power (8–13 Hz). The colors indicate the direction of the contrast (red: first; blue: second). For musicians, no significant differences were observed at stimulus onset or during sound or sound offset (**A**–**C**). For nonmusicians, the parietal area at stimulus onset (**D**), temporal and occipital area at sound (**E**), and occipital area at stim off (**F**) are shown. Statistics: linear mixed-effects model with cluster-based permutation correction (*p* < 0.05). Blue cross lines indicate a pick cluster.

For the source analysis, on the basis of our time-frequency analysis data, we constrained our frequency band of interest to 8–13 Hz, corresponding to the center of the alpha band, as it exhibited the strongest activity (see Section 3.2, Figure 3). The temporal window of interest was defined as the period from the onset to the offset of the auditory speech (Sound), with a fixed duration of 470 ms across all experimental conditions; this window was used for the analyses presented in Figures 4 and 6. To examine the impact of perceptual recalibration on auditory-only trials (serving as a current trial), we segmented each trial into three distinct temporal windows—stimulus onset, sound, and sound offset—for the analyses shown in Figure 7 (see Section 2.2 Stimuli and Figure 1). Alpha power was computed separately for each of these windows to examine dynamic changes across successive processing stages.

Time-frequency analysis within this interval was conducted using the discrete prolate spheroidal sequence (dpss) multitaper method, which computes both the power and cross-spectral density in the alpha frequency range. A spectral smoothing parameter of 4 Hz was applied to optimize the frequency resolution and minimize spectral leakage. Source localization was performed using the DICS beamforming method [44] as implemented in the FieldTrip toolbox, enabling estimation of frequency-specific source power across the full brain volume with high spatial resolution. Precomputed lead fields and head models were utilized to estimate the source power at the median alpha frequency (10.5 Hz). A standard T1-weighted MRI template and a head model based on the boundary element method (BEM), both of which were provided by the FieldTrip toolbox, were used to construct a three-dimensional template grid with 1-cm resolution in Montreal Neurological Institute (MNI) space. Spatial filters were computed individually for each grid location to maximally suppress contributions from all other sources and isolate activity at the target position. Key parameters for the DICS beamformer included 5% regularization of the cross-spectral density matrix, which stabilizes the spatial filter computation by reducing the influence of noise and mitigating issues related to ill-conditioned data. To account for background noise in the estimation of source power, noise projection was enabled, ensuring that the estimated source activity reflected task-related neural responses rather than nonspecific noise. To best capture task-related changes, source power at 10.5 Hz was quantified for both pre-stimulus (−400 to −100 ms) and post-stimulus windows corresponding to each of the temporal segments described above. Alpha power band was extracted voxel-wise from the source grid, and for each voxel, the relative power change was calculated by dividing the change in power by the pre-stimulus power. This normalization controlled for inter-individual differences in baseline oscillatory activity and improved comparability across subjects and conditions. This yielded a normalized power difference defined as follows:

$$Relative\;Power\;Change=\frac{P_{post}-P_{pre}}{P_{pre}}$$

where $P_{pre}$ and $P_{post}$ represent the source power during the pre-stimulus and post-stimulus periods, respectively. This measure provides a normalized index of event-related alpha modulation, allowing direct comparison of dynamic neural responses across different conditions, groups, and analysis window.

### 2.5. Statistical Analysis

#### 2.5.1. Behavioral Data

For the behavioral percentage choice, the main effects of the subject groups (musician vs. nonmusician), the conditions (Aonly-A, Aonly-I, Cong, Incong-A, Incong-I; Cong_T1_-A, Cong_T1_-I, Incong_T1_-A, and Incong_T1_-I) were evaluated using repeated-measures analysis of variance (rmANOVA). We performed this analysis using the fitrm and ranova functions in MATLAB. Post hoc testing was applied using Tukey’s honestly significant difference tests.

#### 2.5.2. Source-Level EEG Data

To investigate the effects of group (musicians vs. nonmusicians) and condition on alpha power in source-localized EEG data, we employed a linear mixed-effects (LME) modeling approach combined with a non-parametric permutation test and cluster-based correction for multiple comparisons. The statistical analyses were conducted using custom MATLAB scripts with functions from FieldTrip [42] and lmeEEG [45]. Alpha power was extracted from source-localized EEG data using the DICS beamforming method. For each voxel, an LME model was fitted (fitlme) to the normalized alpha power data using restricted maximum likelihood estimation, with fixed effects of condition, group, and their interaction, and a random subject effect:

AlphaPower ∼ Condition × Group + (1∣Subject)

Marginal alpha power was computed as the sum of fixed-effect predictions and residuals. Mass univariate regressions yielded t-statistics per voxel and predictor. Statistical significance was assessed via permutation testing (n = 2000), with cluster-based correction for multiple comparisons (cluster-forming threshold: t > 2). Because the analysis was restricted to the alpha frequency band, multiple-comparison correction was performed only across spatial voxels (regions). No region of interest (ROI) was defined a priori; instead, the cluster-based permutation test was applied across the entire source grid to identify significant group differences in a data-driven manner. Permutations were stratified by within-subject or interaction effects as appropriate. The cluster mass statistic was defined as the sum of the t-values within each contiguous suprathreshold voxel. Cluster-level family-wise error (FWE) was controlled at *p*_cluster was <0.05.

To ensure the reliability and stability of the identified topographical clusters, this non-parametric, data-driven framework inherently accounted for spatial relationships between neighboring voxels. This approach reduces the likelihood that individual voxels reach significance by chance and identifies clusters that reflect consistent and robust spatial patterns across participants. In addition, the use of 2000 random permutations ensured convergence of the permutation distribution, confirming the stability of the observed cluster configuration.

Post-hoc tests were used to examine condition effects within groups and group effects within conditions using simplified LME models (n = 2000), with identical cluster-based correction procedures.

#### 2.5.3. Correlation Between Alpha Power and Behavior

To examine the relationship between behavioral performance and source-localized alpha power, we computed Pearson correlations. The behavioral data (percent correct) were categorized by condition and group. Alpha power was extracted from DICS beamforming outputs and averaged across trials and all channels per subject and condition. For each group, condition, and latency, Pearson correlation coefficients and *p* values were calculated between percent correct and alpha power using the corr function on paired subject-level data. Results were considered significant when *p* was <0.05, uncorrected. To account for multiple comparisons across conditions and latencies, false discovery rate (FDR) correction was applied to the full set of *p* values using the mafdr function.

#### 2.5.4. Effect Sizes

To assess effect sizes for group and condition comparisons for behavioral data, a two-way analysis of variance (ANOVA) with effect size estimation was conducted. Effect sizes were calculated using the mes2way function, which computes partial eta-squared effect sizes for each factor and their interaction via a bootstrapping procedure with 2000 iterations. All the data are expressed as the means ± standard errors (SEs) unless otherwise stated.

## 3. Results

### 3.1. Behavioral Analysis of Audio-Visual Information

**Figure 2:**
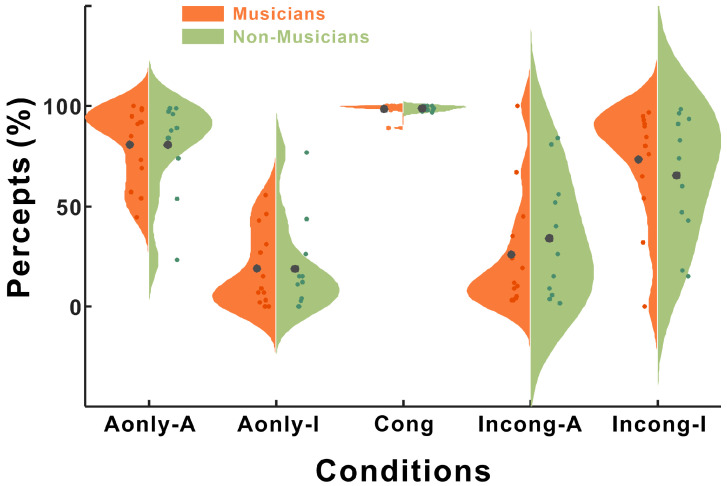
Behavioral responses across the five conditions. Violin plots showing the distribution of percepts (%) for each condition, separately for musicians (orange) and nonmusicians (green). Conditions: Aonly-A = auditory-only trials with auditory responses (/pa/); Aonly-I = auditory-only trials with illusory responses (/ta/); Cong = congruent audiovisual (AV) trials with /pa/ percepts; Incong-A = incongruent AV trials reported as the auditory /pa/ syllable; Incong-I = incongruent AV trials reported as the McGurk illusion (/ta/). The small dots represent individual participants, and the larger gray dots indicate the group means.

Following trial selection (see Methods), the average percentage of trials included in the analysis per participant was as follows: for auditory perception (Aonly-A), 80.77% (±5.29) and 80.65% (±7.0); for illusory perception under the audio-only condition (Aonly-I), 18.88% (±5.38) and 18.73% (±6.96). For the congruent condition (Cong), 98.59% (±0.85) and 98.86% (±0.36) of the trials were included. In the incongruent condition, the percentages were 25.83% (±8.16) and 33.98% (±9.24) for auditory perception (Incong-A) and 73.43% (±8.12) and 65.39% (±9.31) for illusory perception (Incong-I), for the musician and nonmusician groups, respectively (Figure 2).

We performed rmANOVA to examine the main effects of group and condition. The main effect of group was not significantly different (F(1,80) = 0.011, *p* = 0.916, partial η^2^ = 5.098 × 10^−4^), whereas the main effect of condition significantly differed (F(4,80) = 38.69, *p* < 0.001, partial η^2^ = 0.566).

Post hoc analysis revealed significant differences across conditions, highlighting distinct patterns of responses. The comparison involving Aonly-A indicated that this condition resulted in significantly higher values than Aonly-I (*p* < 0.001) and Incong-A (*p* < 0.001) did but significantly lower values than Cong (*p* = 0.002) yielded. No significant difference was found between Aonly-A and Incong-I (*p* = 0.906).

Compared with the other conditions, the congruent condition consistently had significantly greater values for Aonly-I (*p* < 0.001), Incon-I (*p* = 0.002), and Incong-A (*p* < 0.001). In the incongruent conditions, compared with Incong-A, Incong-I yielded significantly lower values (*p* = 0.018) but was comparable to Aonly-A (*p* = 0.906).

### 3.2. Neural Oscillatory Patterns on Audio-Visual Integration

The time-frequency representations of neural activity, averaged across occipito-parietal electrodes identified on the basis of the regions showing the strongest alpha activity in the topographical maps for the five conditions in the musician and nonmusician groups, are shown in Figure 3. In both groups, distinct spectral patterns were observed across conditions, with notable differences in alpha activity detected. On the basis of these observations, further source analysis focusing on alpha power was conducted using DICS. The upper right panels of each condition in Figure 3 display the grand-averaged alpha power across participants during stimulus presentation, calculated as the relative power change compared to the baseline, which reflects the task-induced alpha power. Across all the conditions, both musicians and nonmusicians exhibited negative alpha power.

We examined the effects of group (musicians vs. nonmusicians), condition (Aonly-A, Aonly-I, Cong, Incong-A, and Incong-I), and their interaction on source-localized alpha power using a linear mixed-effects model with a cluster-based permutation test. During the Sound period, significant clusters were identified the main effect of group (*p* = 0.023; peak t = 3.7 at MNI [−50, −70, −10]), for the main effect of condition (*p* < 0.001; peak t = 21.5 at MNI [−10, −60, 40]) and the interaction of group and condition (*p* < 0.001; peak t = 5.21 at MNI [20, −90, −20]).

Post-hoc comparisons for the interaction effect revealed differential patterns of cortical activation between musicians and nonmusicians. Interestingly, compared with musicians in the Cong (*p* < 0.001; peak t = 2.23 at MNI [−10, 30, −20]) and Incong-I (*p* < 0.001; peak t = 2.17 at MNI [−10, 20, 40]) conditions, musicians in the Incong-A condition exhibited less suppressed alpha power (Figure 4a,b), with effects localized to frontal regions. In contrast, nonmusicians displayed lower alpha power in the Incong-A condition than in the Cong (*p* = 0.001; peak t = −2.9 at MNI [60, −30, 50]) and Incong-I (*p* < 0.001; peak t = −2.35 at MNI [60, −30, 40]) conditions (Figure 4d,e), with significant clusters detected in the parietal area.

Notably, under audiovisual incongruence (Cong vs. Incong-I), musicians displayed significantly greater alpha power in the temporal area in the Cong condition than in the Incong-I condition I (*p* < 0.001; peak t = 2.7 at MNI [60, 10, −20], Figure 4c). Nonmusicians, on the other hand, presented greater alpha power in the occipital area in the Incong-I condition than in the Cong condition (*p* = 0.017; peak t = −2.32 at MNI [50, −80, −10]), Figure 4f).

### 3.3. Behavioral Impact of Preceding Trials on Auitory-Only Responses

**Figure 5:**
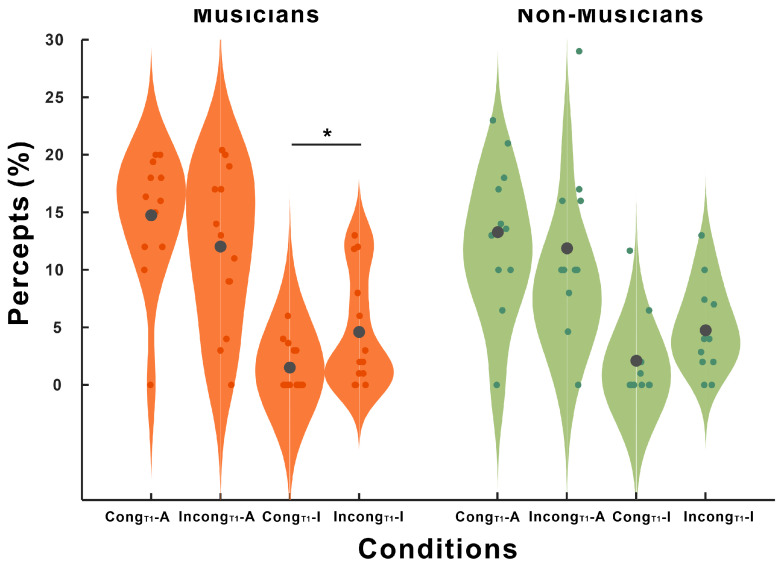
Behavioral responses of prior audiovisual context on auditory-only trials. Violin plots showing the distribution of percepts (%) for the four conditions in the current auditory-only trials, separated by group (orange = musicians; green = nonmusicians). Conditions are labeled by the preceding (T1) audiovisual trial and the current response: Cong_T1_-A = current auditory response after a congruent AV trial; Incong_T1_-A = current auditory response after an incongruent AV trial; Cong_T1_-I = current illusory response after a congruent AV trial; Incong_T1_-I = current illusory response after an incongruent AV trial. The small dots are individual participants; the large gray dots indicate the group means. Note that * indicates a significant difference (*p* < 0.05).

As shown in Figure 2, the percentages of illusory choice in the A-only condition were unexpectedly high in both groups (Mus: 18.88%, non-Mus: 18.73%). This raised the possibility that perceptual bias from the preceding trial might influence subsequent auditory-only processing. We hypothesized that the musicians, due to their enhanced top-down control, would show a smaller recalibration effect (i.e., less influence from incongruent preceding trials) compared to the nonmusicians. To test this hypothesis, we conducted a follow-up analysis examining whether the type of the immediately preceding trial (T1), specifically whether it was a congruent or incongruent audio-visual (AV) condition, modulated responses in the current A-only trial. We excluded an auditory-only condition as a preceding trial to maintain consistent criteria with audio-visual information. These were classified on the basis of the responses to the current Aonly trial as Cong_T1_-A, Incong_T1_-A, Cong_T1_-I, and Incong_T1_-I. The percentage of choices for the four conditions in both groups is shown in Figure 5.

The main effect of group was not significant (F(1,22) = 0.22, *p* = 0.646, partial η^2^ = 0.01). In contrast, a significant main effect of condition was detected (F(3,66) = 21.58, *p* < 0.001, partial η^2^ = 0.495). Subsequent post-hoc comparisons revealed no significant difference between the Cong_T1_-A condition and the Incong_T1_-A condition (*p* = 0.765). However, responses were significantly greater in the Incong_T1_-I condition than in the Cong_T1_-I (*p* = 0.006) condition, and significantly greater in the Incong_T1_-A condition than in the Incong_T1_-I condition (*p* = 0.02).

Although the group × condition interaction did not reach statistical significance (F(3,66) = 4.71, *p* = 0.944, partial η^2^ = 0.06), exploratory post-hoc comparisons revealed within-group differences that may suggest differential sensitivity to prior AV congruency. Specifically, illusory responses to audio-only trials significantly differed in the musician group (*p* = 0.035) but not in the nonmusician (*p* = 0.124) group, depending on whether they were preceded by an incongruent (Incong _T1_-I) or congruent (Cong_T1_-I) AV trial.

### 3.4. Neural Correlates of Preceding Trial Influence on Auditory-Only Responses

We also examined the effects of group (musicians vs. nonmusicians), condition (Cong_T1_-A, Incong_T1_-A, Cong_T1_-I, and Incong_T1_-I), and their interaction on source-localized alpha power using a linear mixed-effects model with a cluster-based permutation test. Unlike the five conditions shown in Figure 4, the four conditions related to auditory-only trials preceded by audiovisual congruency exhibited positive alpha power across all conditions. The linear-mixed effects permutation tests revealed distinct patterns across the four condition comparisons.

For the Cong_T1_-A vs. Incong_T1_-A contrast, no significant differences were found in the musician group (Figure 6A). In contrast, alpha power in the temporal pole was significantly greater in the nonmusician group in the Incong_T1_-A condition than in the Cong_T1_-A condition (*p* = 0.025; peak t = 2.04 at MNI [−30, 20, −40], Figure 6D). For the Cong_T1_-I vs. Incong_T1_-I comparison, no significant differences were observed in the musician group (Figure 6B), whereas the nonmusician group exhibited significantly lower alpha power in the temporal area during the Cong_T1_-I condition than during the Inong_T1_-I condition (*p* < 0.001; peak t = 3.48 at MNI [60, 10, −20], Figure 6E). In the Incong_T1_-A vs. Incong_T1_-I comparison, the musician group exhibited no significant alpha power during the Incong_T1_-I condition compared to Incong_T1_-A condition (Figure 6C). On the other hand, nonmusician group showed greater alpha power in the temporal pole during the Incong_T1_-A condition relative to the Incong_T1_-I condition (*p* < 0.001; peak t = 2.41 at MNI [−30, 20, −40], Figure 6F).

Given the absence of significant differences in musicians for the Cong_T1_-I vs. Incong_T1_-I contrast during the sound period, we further investigated whether musicians process preceding audiovisual information earlier or recalibrate their perception more quickly than nonmusicians do. To examine these temporal dynamics, alpha power was separately analyzed across three time intervals: stimulus onset, sound presentation, and sound offset (Figure 7).

Musicians did not exhibit significant differences across the entire period. In contrast, nonmusicians showed greater alpha power in the Cong_T1_-I condition than in the Incong_T1_-I condition, specifically in occipital areas during stimulus onset (*p* < 0.001; peak t = 2.91 at MNI [20, −70, 40]; Figure 7D), temporal and occipital areas during the sound period (*p* < 0.001; peak t = 3.48 at MNI [60, 10, −20]; Figure 7E), and occipital areas during the sound-off period (*p* = 0.049; peak t = 2.62 at MNI [10, −7,0 −10]; Figure 7F).

### 3.5. Correlations Between Alpha Power and Behavior

**Figure 8:**
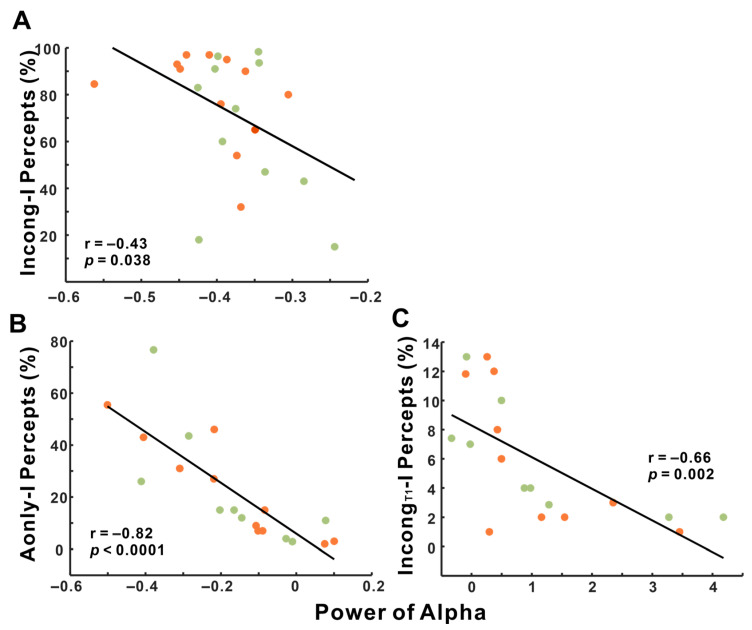
Correlations between source-level alpha power and behavior. Pearson correlations between alpha power (8–13 Hz) during the sound period and the illusory percepts in three conditions: (**A**) incongruent AV trials, (**B**) auditory-only trials reported as illusory, and (**C**) auditory-only trials preceded by incongruent AV trials with illusory responses. Each dot represents a participant (orange = musician, green = nonmusician). The black line is the fit across all the participants.

Correlations analysis examining the relationship between illusory percept rates and alpha power were conducted across all participants, including both musicians and nonmusicians (Figure 8). As shown in Figure 8A, a significant negative correlation was observed between alpha power and illusory percepts for incongruent trials when all participants were considered together (r = −0.43, *p* = 0.038). However, when analyzed separately, neither the musician (r = −0.36, *p* = 0.249) nor the nonmusician groups were correlated (r = −0.42, *p* = 0.203) for this condition. The strong negative correlation between alpha power and illusory percepts in auditory only trials across all participants (r = −0.82, *p* < 0.0001) is shown in Figure 8B, with both musicians and nonmusicians exhibiting significant within-group correlations (musicians: r = −0.91, *p* = 0.0001; nonmusicians: r = −0.73, *p* = 0.02). Similarly, as shown in Figure 8C, a significant negative correlation between alpha power and Incong_T1_-I percept was found in the combined group (r = −0.66, *p* = 0.0002), with both groups individually presenting significant correlations (musicians: r = −0.65, *p* = 0.041; nonmusicians: r = −0.73, *p* = 0.027).

## 4. Discussion

The present study aimed to examine how musical training and preceding audiovisual experiences influence multisensory speech perception, focusing on cortical alpha oscillations. To achieve this goal, we used an experimental design with the McGurk illusion and included both the musician and nonmusician groups. Although no differences were found between the musician and nonmusician groups in terms of overall McGurk susceptibility, we observed notable group differences in the neural dynamics underlying audiovisual integration and perceptual recalibration, highlighting potentially distinct strategies in musicians versus nonmusicians.

### 4.1. Effects of Musical Training

Musical training did not significantly alter behavioral susceptibility to the McGurk illusion. Both groups reported comparable rates of illusory percepts (approximately 73% for musicians and 65% for nonmusicians), aligning with previous studies reporting no expertise-driven differences in illusion strength [9,23]. At the neural level, alpha oscillations were broadly suppressed during audio-visual processing compared with baseline across all conditions, which aligns with alpha suppression as a neural marker for active sensory processing and attentional engagement [31,38,46].

Despite the absence of group differences in behavior, EEG source analysis revealed distinct neural patterns. Nonmusicians displayed stronger alpha suppression in the parietal alpha band in the Incong-A condition than in the Cong and Incong-I conditions. Parietal alpha suppression is often interpreted as indicative of enhanced sensory-driven, bottom-up processing and attention toward the resolution of audiovisual conflict in less-trained individuals [32,33].

In contrast, musicians exhibited more suppressed alpha power in frontal areas during the Cong or Incong-I conditions compared to the Incong-A condition. Frontal alpha oscillations have been closely associated with top-down control [35,47,48], reflecting attentional modulation and suppression of irrelevant sensory information. Thus, one interpretation is that the greater frontal alpha power suppression might reflect an enhanced top-down neural strategy for integrating audiovisual information. This distinction suggests that musical training may enhance the brain’s ability to adaptively regulate alpha oscillations according to task demands [49,50]. Specifically, this enhanced top-down control is consistent with research indicating that musical training strengthens connectivity between sensory higher-order control areas, facilitating the dynamic gating of sensory information [49,51,52].

An alternative explanation is that this frontal–parietal split reflects a more general difference in cognitive load or strategy, rather than a mechanism specific to audiovisual integration [53]. Within a predictive coding framework [54], musicians’ extensive audio-visual integration experience may have strengthened integrative expectation. When faced with incongruent stimuli, these priors are violated, generating prediction errors that recruit top-down control mechanisms to resolve conflict. Nonmusicians, perhaps lacking such strong expectations, may engage more in the bottom-up (parietal) processing.

### 4.2. Perceptual History

We found that perceptual history from preceding trials significantly modulated responses in auditory-only conditions. Specifically, illusory responses to the auditory-only trials were significantly dependent on the preceded congruency (Cong_T1_-I vs. Incong_T1_-I). This result is consistent with recalibration accounts in which recent perceptual conflict shifts cue weighting to balance stability and sensitivity [16,26].

This interpretation, however, also suggests a crucial ambiguity regarding the role of attention. One possibility is that the absence of visual support in auditory-only trials may lessen attentional engagement with the current stimulus, thereby increasing uncertainty, leading to higher illusory percepts following incongruent stimuli. An alternative possibility is that the effect is not a specific recalibration, but rather a more general attentional load or working memory updating following conflict preceding trial [53].

Although the group × condition interaction did not reach statistical significance, exploratory analyses offered a nuanced picture. Within-group tests exhibited significant differences in the musician but not in the non-musician groups. This discrepancy— non-significant interaction despite a significant trend within the musician group—leads us to consider that the overall null effect might be related to insufficient statistical power. The lack of significant finding within nonmusicians may stem from the mixed trials of auditory and illusory responses in the incongruent priors, because low trial numbers prevented the illusory-only preceded trial filtering used by Lüttke et al. from revealing stronger biases [17]. Despite this ambiguity, correlation analyses revealed that lower alpha power corresponded with stronger behavioral carryover (Figure 8C), supporting an inhibitory-gating account of recalibration [38,55]. Alpha suppression may thus index reduced inhibition, allowing prior sensory traces to bias current perception.

In terms of neural activity, nonmusicians presented greater alpha power in the temporal area for Incong_T1_-A than for Cong_T1_-A or Incong_T1_-I. A plausible interpretation is that this elevated alpha reflects inhibitory gating to suppress residual visual influence from the preceding incongruent trial, thereby stabilizing an auditory based percept [28,30,38,56]. We expect this interpretation would be clearer if preceding trials were response-filtered (e.g., keeping only incongruent trials with illusory responses in the preceded trials), as reported in the study by Lüttke et al. [17]; limited trial counts prevented that analysis here. Additionally, nonmusicians exhibited greater temporal and occipital alpha power for Cong_T1_-I than for Incong_T1_-I. This result suggests that congruent histories establish an audio-visual prior that suppresses integration areas when visual input is absent. After incongruent histories, lower alpha power might reflect a more stimulus-driven engagement of audiovisual circuitry. This is consistent with a plausible explanation is that prediction-driven processing generates the illusion while suppressing competing sensory evidence in occipitotemporal cortex [28,54,57].

In musicians, however, this pattern differed. The “same-response, different-history” comparisons—Cong_T1_-A versus Incong_T1_A and Cong_T1_-I/ Incong_T1_-I—revealed no significant areas for alpha power, indicating that once the current percept is determined, cortical dynamics are relatively insensitive to whether the preceding trial was congruent or incongruent, implying reduced reliance on immediate context. Notably, this neural pattern diverged from behavior in the Cong_T1_-I vs. Incong_T1_-I comparison, which showed a significant difference. To probe this neural–behavioral mismatch, we compared Cong_T1_-I vs. Incong_T1_-I across three time windows (stim onset, sound, and sound off). This temporal decomposition tests whether recalibration is time-specific rather than sustained [30,34]. Nonmusicians exhibited sustained occipital alpha differences between Cong_T1_-I than for Incong_T1_-I throughout all periods, suggesting ongoing context-driven gating and continuous model updating to resolve sensory prediction errors and recalibrate the internal model. Musicians, in contrast, showed no significant clusters emerged across time windows. This absence may indicate that relevant inhibitory adjustments occur earlier or later than our analysis windows or decision formation, perhaps involving more on predictive, timing-sensitivity circuitry rather than sustained oscillatory modulation [38].

Collectively, these findings offer a novel contribution to theoretical models of perceptual recalibration [15,17]. While these models often treat recalibration as a relatively uniform, sensory-driven process, our results provide clear neural evidence that this process is modulated by expertise. This is particularly noteworthy given that this link is not universal; for instance, musical training was recently found to not affect temporal recalibration [51]. Our study thus extends these models by demonstrating that the neural signature of perceptual history is not fixed but is malleable and highly dependent on an individual’s perceptual training.

### 4.3. Limitations and Future Directions

Several limitations should be noted. The small sample size (n = 24) may have limited the statistical power for detecting subtle behavioral interactions, where marginal trends suggest potential underpowered insights into perceptual history influences [5]. Reliance on self-reported musical training without objective measures (e.g., standardized proficiency tests) introduces variability, and the cross-sectional design precludes causal conclusions [58]. The use of a single speaker and limited syllables also reduces generalizability and may have attenuated behavioral group differences [3,4,8,16]. Furthermore, although DICS beamforming with 64-channel EEG provided source-localized alpha power estimates, the limited spatial resolution might constrain the precision of regional interpretations; these results should be interpreted cautiously and, ideally, validated with higher-density EEG or multimodal imaging such as functional magnetic resonance imaging (fMRI) for better anatomical specificity [44,59]. Finally, the exclusive inclusion of right-handed participants controlled for brain lateralization confounds but limits generalizability, as handedness is strongly associated with the laterality of sensorimotor functional connectivity [60].

Future directions could address these issues by employing larger cohorts and diverse stimuli. Exploring other frequency bands (e.g., theta) and connectivity measures, such as phase-locking value (PLV) or graph theory could clarify the underlying mechanisms, revealing how alpha interacts with network dynamics in recalibration processes [61,62]. Finally, it is also important to explore broader applications, such as how our findings on expertise-related top-down mechanisms could inform cognitive rehabilitation strategies or aging effects [63]. These combined approaches would provide a more comprehensive understanding of expertise and history in multisensory speech perception.

## 5. Conclusions

This study demonstrates the complex interplay of perceptual expertise and cognitive history in shaping audiovisual speech perception. While behavioral outcomes in McGurk susceptibility were similar, the underlying neural dynamics diverged. Musical training is associated with frontal alpha modulation, a finding consistent with enhanced top-down control or more efficient predictive processing strategies. Nonmusicians appear to rely more on sensory-driven bottom-up processes and show neural signatures consistent with stronger perceptual recalibration effects based on preceding experiences. These findings advance our understanding of the neural dynamics underpinning the McGurk illusion and multisensory speech integration, highlighting the value of interpreting such dynamics within broader theoretical frameworks, such as predictive coding, and considering the potential role of cognitive factors alongside implications for models of perceptual learning and sensory processing.

## Figures and Tables

**Table 1:** Characteristics of musicians.

Subjects	First Instrument	Age Began Musical Training (yrs)	Secondary Instrument	Age Began Musical Training (yrs)	Years of Musical Training
Mus 1	Korean traditional Vocal	14			20
Mus 2	Piano	9			12
Mus 3	Piano	9			11
Mus 4	Piano	6	Vocal	17	20
Mus 5	Bass guitar	20			16
Mus 6	Piano	6	Cello	25	19
Mus 7	Piano	5	Vocal	23	25
Mus 8	Haegeum	16	Vocal	24	10
Mus 9	Piano	8	Clarinet	16	20
Mus 10	Drum	17			12
Mus 11	Guitar	10			10
Mus 12	Violin	3	Piano	10	13
Mus 13	Guitar	13			13

## Data Availability

The data presented in this study are available in OSF (Open Science Framework) at: https://osf.io/b3c5j/overview, accessed on 16 November 2025.

## References

[1] Samuel A.G. (2011). Speech perception. Annu. Rev. Psychol..

[2] Mcgurk H., Macdonald J. (1976). Hearing lips and seeing voices. Nature.

[3] Nath A.R., Beauchamp M.S. (2012). A neural basis for interindividual differences in the McGurk effect, a multisensory speech illusion. NeuroImage.

[4] Strand J.F., Cooperman A., Rowe J., Simenstad A. (2014). Individual Differences in Susceptibility to the McGurk Effect: Links With Lipreading and Detecting Audiovisual Incongruity. J. Speech Lang. Hear. Res..

[5] Alsius A., Paré M., Munhall K.G. (2018). Forty Years after Hearing Lips and Seeing Voices: The McGurk Effect Revisited. Multisens. Res..

[6] Sakamoto S., Mishima H., Suzuki Y. (2012). Effect of Consonance between Features and Voice Impression on the McGurk Effect. Interdiscip. Inf. Sci..

[7] van Wassenhove V., Grant K.W., Poeppel D. (2007). Temporal window of integration in auditory-visual speech perception. Neuropsychologia.

[8] Schwartz J.L. (2010). A reanalysis of McGurk data suggests that audiovisual fusion in speech perception is subject-dependent. J. Acoust. Soc. Am..

[9] Proverbio A.M., Massetti G., Rizzi E., Zani A. (2016). Skilled musicians are not subject to the McGurk effect. Sci. Rep..

[10] Grant K.W., Seitz P.F. (1998). Measures of auditory–visual integration in nonsense syllables and sentences. J. Acoust. Soc. Am..

[11] Navarra J., Alsius A., Soto-Faraco S., Spence C. (2010). Assessing the role of attention in the audiovisual integration of speech. Inf. Fusion.

[12] Palmer T.D., Ramsey A.K. (2012). The function of consciousness in multisensory integration. Cognition.

[13] Berger C.C., Ehrsson H.H. (2013). Mental imagery changes multisensory perception. Curr. Biol..

[14] Tuomainen J., Andersen T.S., Tiippana K., Sams M. (2005). Audio-visual speech perception is special. Cognition.

[15] Lüttke C.S., Ekman M., Van Gerven M.A.J., De Lange F.P. (2016). McGurk illusion recalibrates subsequent auditory perception. Sci. Rep..

[16] Magnotti J.F., Lado A., Zhang Y., Maasø A., Nath A., Beauchamp M.S. (2024). Repeatedly experiencing the McGurk effect induces long-lasting changes in auditory speech perception. Commun. Psychol..

[17] Lüttke C.S., Pérez-Bellido A., de Lange F.P. (2018). Rapid recalibration of speech perception after experiencing the McGurk illusion. R. Soc. Open Sci..

[18] Micheyl C., Delhommeau K., Perrot X., Oxenham A.J. (2006). Influence of musical and psychoacoustical training on pitch discrimination. Hear. Res..

[19] Psarris G., Eleftheriadis N., Sidiras C., Sereti A., Iliadou V.M. (2024). Temporal resolution and pitch discrimination in music education: Novel data in children. Eur. Arch. Oto Rhino Laryngol..

[20] Mishra S.K., Panda M.R., Herbert C. (2014). Enhanced auditory temporal gap detection in listeners with musical training. J. Acoust. Soc. Am..

[21] Hyde K.L., Lerch J., Norton A., Forgeard M., Winner E., Evans A.C., Schlaug G. (2009). Musical training shapes structural brain development. J. Neurosci..

[22] Musacchia G., Sams M., Skoe E., Kraus N. (2007). Musicians have enhanced subcortical auditory and audiovisual processing of speech and music. Proc. Natl. Acad. Sci. USA.

[23] Lee H.H., Groves K., Ripollés P., Carrasco M. (2024). Audiovisual integration in the McGurk effect is impervious to music training. Sci. Rep..

[24] Politzer-Ahles S., Pan L. (2019). Skilled musicians are indeed subject to the McGurk effect. R. Soc. Open Sci..

[25] Van der Burg E., Alais D., Cass J. (2018). Rapid recalibration to audiovisual asynchrony follows the physical—Not the perceived—Temporal order. Atten. Percept. Psychophys..

[26] Fritsche M., Mostert P., de Lange F.P. (2017). Opposite Effects of Recent History on Perception and Decision. Curr. Biol..

[27] Scheliga S., Kellermann T., Lampert A., Rolke R., Spehr M., Habel U. (2023). Neural correlates of multisensory integration in the human brain: An ALE meta-analysis. Rev. Neurosci..

[28] Park H., Kayser C. (2019). Shared neural underpinnings of multisensory integration and trial-by-trial perceptual recalibration in humans. Elife.

[29] Romero Y.R., Keil J., Balz J., Niedeggen M., Gallinat J., Senkowski D. (2016). Alpha-band oscillations reflect altered multisensory processing of the McGurk illusion in Schizophrenia. Front. Hum. Neurosci..

[30] Keil J., Senkowski D. (2018). Neural Oscillations Orchestrate Multisensory Processing. Neuroscientist.

[31] Lange J., Keil J., Schnitzler A., van Dijk H., Weisz N. (2014). The role of alpha oscillations for illusory perception. Behav. Brain Res..

[32] Lum J.A.G., Barham M.P., Hyde C., Hill A.T., White D.J., E Hughes M., Clark G.M. (2024). Top-down and bottom-up oscillatory dynamics regulate implicit visuomotor sequence learning. Cereb. Cortex.

[33] Mercier M.R., Molholm S., Fiebelkorn I.C., Butler J.S., Schwartz T.H., Foxe J.J. (2015). Neuro-oscillatory phase alignment drives speeded multisensory response times: An electro-corticographic investigation. J. Neurosci..

[34] MacAluso E., Noppeney U., Talsma D., Vercillo T., Hartcher-O’Brien J., Adam R. (2016). The Curious Incident of Attention in Multisensory Integration: Bottom-up vs. Top-down. Multisensory Res..

[35] Misselhorn J., Friese U., Engel A.K. (2019). Frontal and parietal alpha oscillations reflect attentional modulation of cross-modal matching. Sci. Rep..

[36] Friese U., Daume J., Göschl F., König P., Wang P., Engel A.K. (2016). Oscillatory brain activity during multisensory attention reflects activation, disinhibition, and cognitive control. Sci. Rep..

[37] Bertoni T., Noel J.-P., Bockbrader M., Foglia C., Colachis S., Orset B., Evans N., Herbelin B., Rezai A., Panzeri S. (2025). Pre-movement sensorimotor oscillations shape the sense of agency by gating cortical connectivity. Nat. Commun..

[38] Jensen O., Mazaheri A. (2010). Shaping functional architecture by oscillatory alpha activity: Gating by inhibition. Front. Hum. Neurosci..

[39] Klein C., Diaz Hernandez L., Koenig T., Kottlow M., Elmer S., Jäncke L. (2016). The Influence of Pre-stimulus EEG Activity on Reaction Time During a Verbal Sternberg Task is Related to Musical Expertise. Brain Topogr..

[40] Kausel L., Zamorano F., Billeke P., Sutherland M.E., Alliende M.I., Larrain-Valenzuela J., Soto-Icaza P., Aboitiz F. (2024). Theta and alpha oscillations may underlie improved attention and working memory in musically trained children. Brain Behav..

[41] Zhou Y.J., Iemi L., Schoffelen J.M., de Lange F.P., Haegens S. (2021). Alpha oscillations shape sensory representation and perceptual sensitivity. J. Neurosci..

[42] Oostenveld R., Fries P., Maris E., Schoffelen J.M. (2011). FieldTrip: Open source software for advanced analysis of MEG, EEG, and invasive electrophysiological data. Comput. Intell. Neurosci..

[43] Delorme A., Makeig S. (2004). EEGLAB: An open source toolbox for analysis of single-trial EEG dynamics including independent component analysis. J. Neurosci. Methods.

[44] Gross J., Kujala J., Hämäläinen M., Timmermann L., Schnitzler A., Salmelin R. (2001). Dynamic imaging of coherent sources: Studying neural interactions in the human brain. Proc. Natl. Acad. Sci. USA.

[45] Visalli A., Montefinese M., Viviani G., Finos L., Vallesi A., Ambrosini E. (2024). lmeEEG: Mass linear mixed-effects modeling of EEG data with crossed random effects. J. Neurosci. Methods.

[46] Klimesch W., Sauseng P., Hanslmayr S. (2007). EEG alpha oscillations: The inhibition-timing hypothesis. Brain Res. Rev..

[47] Wang C., Rajagovindan R., Han S.M., Ding M. (2016). Top-down control of visual alpha oscillations: Sources of control signals and their mechanisms of action. Front. Hum. Neurosci..

[48] Wöstmann M., Alavash M., Obleser J. (2019). Alpha oscillations in the human brain implement distractor suppression independent of target selection. J. Neurosci..

[49] Gray R., Sarampalis A., Başkent D., Harding E.E. (2022). Working-Memory, Alpha-Theta Oscillations and Musical Training in Older Age: Research Perspectives for Speech-on-speech Perception. Front. Aging Neurosci..

[50] López-Madrona V.J., Trébuchon A., Bénar C.G., Schön D., Morillon B. (2024). Different sustained and induced alpha oscillations emerge in the human auditory cortex during sound processing. Commun. Biol..

[51] O’Donohue M., Lacherez P., Yamamoto N. (2022). Musical training refines audiovisual integration but does not influence temporal recalibration. Sci. Rep..

[52] MacLean J., Stirn J., Bidelman G.M. (2025). Alpha-Band Brain Activity Shapes Online Perceptual Learning of Concurrent Speech Differentially in Musicians vs. Nonmusicians. Eur. J. Neurosci..

[53] Jiang Y., Qiao R., Shi Y., Tang Y., Hou Z., Tian Y. (2023). The effects of attention in auditory–visual integration revealed by time-varying networks. Front. Neurosci..

[54] Arnal L.H., Giraud A.L. (2012). Cortical oscillations and sensory predictions. Trends Cogn. Sci..

[55] Klimesch W. (2012). Alpha-band oscillations, attention, and controlled access to stored information Open access under CC BY-NC-ND license. Trends Cogn. Sci..

[56] Alpert G.F., Hein G., Tsai N., Naumer M.J., Knight R.T. (2008). Temporal characteristics of audiovisual information processing. J. Neurosci..

[57] Foxe J.J., Snyder A.C. (2011). The role of alpha-band brain oscillations as a sensory suppression mechanism during selective attention. Front. Psychol..

[58] Moreno S., Bidelman G.M. (2014). Examining neural plasticity and cognitive benefit through the unique lens of musical training. Hear. Res..

[59] Van Veen B.D., Van Drongelen W., Yuchtman M., Suzuki A. (1997). Localization of brain electrical activity via linearly constrained minimum variance spatial filtering. IEEE Trans. Biomed. Eng..

[60] Tomasi D., Volkow N.D. (2024). Associations between handedness and brain functional connectivity patterns in children. Nat. Commun..

[61] Fernández L.M., Macaluso E., Soto-Faraco S. (2017). Audiovisual integration as conflict resolution: The conflict of the McGurk illusion. Hum. Brain Mapp..

[62] Roa Romero Y., Senkowski D., Keil J. (2015). Early and late beta-band power reflect audiovisual perception in the McGurk illusion. J. Neurophysiol..

[63] Tragantzopoulou P., Giannouli V. (2025). A Song for the Mind: A Literature Review on Singing and Cognitive Health in Aging Populations. Brain Sci..

